# Cognitive interviewing methodology in the development of a pediatric item bank: a patient reported outcomes measurement information system (PROMIS) study

**DOI:** 10.1186/1477-7525-7-3

**Published:** 2009-01-23

**Authors:** Debra E Irwin, James W Varni, Karin Yeatts, Darren A DeWalt

**Affiliations:** 1 Department of Epidemiology, University of North Carolina at Chapel Hill, Chapel Hill, NC, USA; 2 Department of Pediatrics, College of Medicine, Department of Landscape Architecture and Urban Planning, College of Architecture, Texas A&M University College Station, Texas, USA; 3 Division of General Medicine and Clinical Epidemiology, Cecil G. Sheps Center for Health Services Research, University of North Carolina at Chapel Hill, Chapel Hill, North Carolina, USA

## Abstract

**Background:**

The evaluation of patient-reported outcomes (PROs) in health care has seen greater use in recent years, and methods to improve the reliability and validity of PRO instruments are advancing. This paper discusses the cognitive interviewing procedures employed by the Patient Reported Outcomes Measurement Information System (PROMIS) pediatrics group for the purpose of developing a dynamic, electronic item bank for field testing with children and adolescents using novel computer technology. The primary objective of this study was to conduct cognitive interviews with children and adolescents to gain feedback on items measuring physical functioning, emotional health, social health, fatigue, pain, and asthma-specific symptoms.

**Methods:**

A total of 88 cognitive interviews were conducted with 77 children and adolescents across two sites on 318 items. From this initial item bank, 25 items were deleted and 35 were revised and underwent a second round of cognitive interviews. A total of 293 items were retained for field testing.

**Results:**

Children as young as 8 years of age were able to comprehend the majority of items, response options, directions, recall period, and identify problems with language that was difficult for them to understand. Cognitive interviews indicated issues with item comprehension on several items which led to alternative wording for these items.

**Conclusion:**

Children ages 8–17 years were able to comprehend most item stems and response options in the present study. Field testing with the resulting items and response options is presently being conducted as part of the PROMIS Pediatric Item Bank development process.

## Background

The Patient Reported Outcomes Measurement Information System (PROMIS) project, a National Institute of Health Roadmap for Medical Research initiative, was developed to advance the science and application of patient-reported outcomes (PRO) in chronic diseases [1]. The process of developing item banks for PROMIS includes literature review, focus groups, and individual cognitive interviews [2-4]. Among the qualitative methods, cognitive interviewing allows direct input from respondents on the item content, format, and understandability. This method has emerged as an essential component in the development of a number of standardized measures [5-7].

The cognitive interviewing methodology for PROMIS was designed to elicit input from respondents on all items under consideration for the PROMIS item bank [3]. The pediatric cognitive interviewing methodology followed the general principles of the PROMIS Network [3], with the necessary adaptations required for children as young as 8 years of age, relying in part on the cognitive interviewing methodology utilized in the development of the PedsQL™ instruments [8] and the work of Willis [9].

The cognitive interviewing methodology is designed to assess the cognitive processes underlying respondents' comprehension and generation of answers to questionnaire items within an information processing conceptual model [10]. The intent of cognitive interviewing is to determine what the respondent thinks or comprehends a particular item is asking (what do specific words and phrases in the item stem mean to the respondent); the processes used by the respondent to retrieve relevant information from autobiographical memory; the decision or judgment processes used to conceive an answer; and the process of formulating a response to the item stem [10-13].

Although there are two major types of cognitive interviewing methods (think-aloud and respondent debriefing), the PROMIS cognitive interviews employed the respondent debriefing technique [7]. In this technique, after a participant completes the questionnaire, an interviewer probes for specific information on what types of difficulties respondents experienced while completing the items, and the basis for the response for each item [9]. Cognitive probes elicit information regarding the clarity and rationale of the directions, the meaning of the items, the appropriateness of the response choices, and overall comments on the relevance and complexity of the questionnaire [12,13].

The primary objective of this study was to conduct cognitive interviews with children and adolescents to gain feedback on items measuring physical functioning, emotional health, social health, fatigue, pain, and asthma-specific symptoms.

## Methods

### Item development

The PROMIS Pediatrics project focused on the development of PRO item banks across several health domains for youth ages 8–17 years. Initially, PROMIS focused on the measurement of generic health domains that are important across a variety of illnesses, including physical function, pain, fatigue, emotional distress, and social function [2]. Since asthma is the most common chronic disease of childhood, and PRO measurement is an essential component of evaluation of outcomes for children with asthma [14-16], asthma was an excellent chronic condition for the initial development of the PROMIS pediatrics disease specific item bank.

The PROMIS item bank was developed using a strategic item generation methodology. A series of focus groups were conducted to generate themes and domains [4]; a literature review was conducted to identify existing pediatric health questionnaires; and discussions with health care and research personnel (including physicians, psychologists, social workers, epidemiologists and nurses) were utilized to identify an initial item pool of over 3345 items. These items were "binned" (i.e., items were classified into domains according to their content) and "winnowed" (items were eliminated that either lacked face validity for the domain or were very similar to a more ideally worded item) [2,3] by the PROMIS pediatric project team. Items were rewritten or modified to adhere to a set of formatting requirements accepted by the PROMIS development team (e.g., use of past tense, 7 day recall period, standard response options (see Table 1 for response options utilized)). Cognitive interviews were conducted on the resulting 318 items across 6 domains, after which 35 items were revised and underwent a second round of cognitive interviews. The final item set contained 293 items across 6 domains (Physical Function = 70 items; Emotional Health = 49 items; Social Health = 74 items; Fatigue = 39 items; Pain = 27 items; Asthma = 34 items).

**Table 1:** Item response options

**Frequency**
Never
Almost Never
Sometimes
Often
Almost Always
**Difficulty (or interference)**
With no trouble
With a little trouble
With some trouble
With a lot of trouble
Not able to do

**Numeric Rating: **0–10

**# of Days**: 0,1,2,3,4,5,6,7

### Participants

To participate in the cognitive interviews at The Children's Hospital at Scott and White (S&W) and the University of North Carolina (UNC), participants needed to meet the following criteria: between the ages of 8 and 17 years inclusive; speak and read English; provide informed assent prior to study entry; and provide parent or guardian informed consent. We also recruited children with asthma to review all domain items and asthma-specific items. Participants were not eligible for the study if they had any concurrent medical, psychiatric or cognitive conditions that, in the investigator's opinion, would interfere with participation in this study.

Purposive sampling was used to recruit a total of 28 children and adolescents from the UNC (6 with asthma; 22 without asthma) hospital and community clinics and 37 children and adolescents from the general pediatric clinic at S&W (16 with asthma; 21 without asthma), who participated in the first round of cognitive interviews. For the second round of cognitive interviews, 18 children and adolescents from S&W and 5 children from UNC participated (11 of these 23 participated in first round interviews). Table 2 lists the demographic characteristics of the first round cognitive interview participants from each site. For each domain questionnaire, the cognitive interview sample included at least 2 children 8 or 9 years of age, 1 adolescent between 13 and 18 years, 2 children of non-white ethnicity, and 1 child of white/Caucasian ethnicity. These categories were not exclusive. For example, a Latina girl age 8 would fulfill both the racial/ethnic requirement and the age requirement.

**Table 2:** Participant demographics and clinical characteristics for first round cognitive interviews

	**NC (N = 28 (%)**	**TX (N = 37 (%)**
***Gender ***– Male	14 (50)	19 (51)

***Age***		
8–9 years	11 (39)	13 (35)
10–12 years	8 (29)	16 (43)
13–17 years	9 (32)	8 (22)

***Grade Completed in School***		
1^st ^or less	0	1 (3)
2^nd ^– 5^th^	20 (71)	24 (65)
6^th ^– 11^th^	8 (29)	12 (32)

Reading Level*		
2^nd ^– 5^th^	7 (25)	14 (38)
6^th ^– 8^th^	14 (50)	8 (22)
High School	5 (18)	9 (24)
Post- High School	2 (7)	5 (14)
Missing	0	1 (2)

***Race***		
Caucasian	19 (68)	28 (76)
African American	5 (17)	2 (5)
Asian	3 (11)	0
Other – Mixed	1 (4)	7 (19)

***Ethnicity ***– Hispanic	2 (7)	10 (27)

***Guardian Status***		
Divorced	6 (21)	2 (5)
Separated	2 (7)	5 (14)
Married	15 (54)	25 (68)
Never married	5 (18)	4 (11)
Living with partner	0	1 (2)

***Guardian Education Status***		
Advanced degree	6 (21)	7 (19)
College	18 (65)	5 (13)
Some college/AA	0	21 (57)
High School	4 (14)	4 (11)

***Guardian Occupation***		
Full-Time Employed	16 (57)	27 (73)
Homemaker	7 (25)	2 (7)
Part Time Employed	3 (11)	6 (16)
Unemployed	2 (7)	0
Full time student	0	1 (2)
FT emp & PT student	0	1 (2)

***Asthma***	6 (21)	16 (43)

*based on WRAT score

### Recruitment procedures

At both UNC and the S&W, potential participants were identified through review of clinic appointment books. A research assistant then mailed an informational letter to the child's parent to inform them about the study. Those who were interested in participating contacted the study coordinator to schedule their interview time. If the child was deemed eligible to participate in the cognitive interview and the parents agreed to allow their child to participate, they were scheduled for an interview date. At the time of the interview, a trained research assistant obtained parental informed consent and the children signed an assent document. All child participants received a $25 gift card in return for their time and effort. Children were allowed to take a break or end the interview at any time, although no children ended the interview prematurely. The study protocols were approved by the institutional review boards at UNC (#05-1431) and at S&W (#05-0077).

### Cognitive interviewing process

The interviewers utilized for this study underwent an extensive training session (16 hours) that included general information on cognitive interview theory and procedures, as well as pediatric specific procedures. Interviewers were graduate students in social work or research nurses all who had experience working with children in pediatric research settings. All interviewers were trained by a pediatric psychologist with extensive experience in children's therapy and qualitative questionnaire development. Interviews were conducted in a comfortable environment and breaks were offered for the children.

We applied a sampling scheme that allowed each participant to be interviewed on approximately 30 items rather than all 318 items. Each child evaluated items from only one or two domains and only one response scale. By this method, all items in the bank were reviewed by at least 5 participants (59% of items were reviewed by 5 participant; 34% were reviewed by 6 participants; 7% were reviewed by 7 participants) meeting the target demographic characteristics outlined above (see Participants Section). During the cognitive interviews, participants were asked to provide verbal open-ended feedback on each item regarding response categories, time frame, item interpretation and overall impression of domain content and coverage.

Parents were asked to complete a sociodemographic form which contained information regarding the child's age, gender, ethnicity, living situation, and chronic health condition(s) as well as the parent/guardian's employment and education. Parents of children with asthma also completed an asthma form, which contained information about the number of days and nights in the previous week the child had coughing, wheezing, or shortness of breath, the number of times in the previous week the child used rescue medication, and the types of medications the child was taking. These demographic characteristics are described in Table 2.

Other than the children with asthma who underwent the cognitive interview on the asthma-specific item set, participants were randomly assigned to receive an item set (approximately 30 items) selected from one of the domains. Prior to the cognitive interview, participants completed an item set through paper and pencil administration. A research assistant trained in cognitive interviewing techniques then reviewed each item stem and item response with the child and began the interview using standardized questions (see Table 3) for each item. A subset of participants were asked questions about preference of item tense (past vs. present). The participant's comprehension or interpretation of the item along with their preferences on recall options and recall time period was elicited. All participant answers were recorded on a computerized spreadsheet. At the end of the interview, participants completed the Wide Range Achievement Test-3 Reading Subtest (WRAT) as a gross measure of reading ability [17]. Interviews were also audio-taped to ensure accuracy of interviewer notes.

**Table 3:** Cognitive interview questions

**Directions**
How would you make the directions more clear/easy to understand?
What does "in the past 7 days" mean to you?
When you see "the last 7 days", what days did you include?
**Items**
In your own words, what do you think this question is asking?
What does this question mean to you? What did you think of when answering this question?
Was this question easy to understand? Are there any specific words that are difficult to understand?
How would you change the words to make it more clear?
Was this item hard to answer? If yes, why?
How did you choose your answer?

**Domains**
In your own words, what do you think this group of questions is asking about?
How do you think these items are related?
Are there any questions that don't belong in this group?

**Response Choices**
What do you think about the response choices?
How would you make the response choices clearer or easier to understand?

**Overall Assessment**
Are there things that we forgot to ask about that you think are important?
Overall thoughts/opinions of the questionnaire?
Anything you would change in the questionnaire as a whole?

### Data analysis and item revision

After each interview, project personnel completed a summary statement for each item and the child's comments. After completing all initial cognitive interviews for an item, project personnel compiled reports that included all comments for an item. The item development team then reviewed all of the comments to determine issues with formatting, item comprehension, instructions, tense, and response options (see Table 4). Items deemed problematic by two or more children of any age were revised for clarity. Other items similar to those revised after the initial interview process were also changed by project personnel to maintain consistency across item stems or wording. In all, 35 items were revised as a result of the first round of cognitive interviews.

**Table 4:** Common issues identified by participants in first round of interviews

**General Formatting Issues**	
Make the words larger	
	
**Issues with Instructions**	
Put recall period in bold type	
Instructions are too long	
Young children didn't understand the words "questionnaire" or "accurate"	
	
**Item Comprehension Issues: Word Meaning**	
***Problematic Word***	***Suggested Change***
"clothing drawers"	"dresser drawers"
"irritable"	"grumpy", "cranky"
"worry"	"scared"
"stressed"	"mad", "upset"
"exhausted"	"tired"
"how severe"	"how bad"
"social activities"	"activities with friends"
"ER"	"emergency room"
"grumpy"	"mad", "angry"
"rely"	"trust"
"furious"	"angry", "mad"
"frustrated"	"grouchy", "mad"
"frightened"	"scared", "afraid"
"snaps" (i.e., shirt snaps)	"zipper", "button"
	
**Item Comprehension Issues: Vague/Ambiguous Words/Phrases**	
***ProblematicWords/Phrases***	***Explanation***
"activities"	Could mean sports or hobbies (i.e., crafts)
"clothes"	Could mean pants, shirts, or both
"walk"	Could be a block or a mile
"hard to have fun"	Doesn't specify if it's hard due to physical or emotional issues
"did things"	Isn't specific. What kinds of things?
"go out"	Could mean going outside (i.e., to play) or going out with family/friends (i.e., to dinner)
"relationships"	Could mean relationships with friends, family, teachers, or others
"others"	Could mean friends, family, teachers, strangers, or others
"I felt like I did everything badly"	Unclear if it is due to poor performance or if they got in trouble
"I felt so bad that I didn't want to do anything"	Unclear if "bad" referred to physical health, guilt/shame, or low self-esteem.
"feel terrible"	Could mean physically or emotionally
	
**Issues with Item Tense**	
Past tense items were preferred over present tense items	
	
**Misc. Issues**	
Assistive device items (i.e., questions about using a walker or wheelchair) didn't apply to a large number of children	

Note: All issues in above table were identified by at least 2 children

To ensure comprehension of the 35 revised items, a second set of cognitive interviews was conducted. Project personnel then reviewed the revised items and participants' responses from the second review. Items that continued to be problematic to research participants after the second round were eliminated from the item bank. Table 5 shows the 22 items that were retained in the final item bank and revised after the second round of cognitive interviews, along with the reasons for revising the items.

**Table 5:** PROMIS pediatric revised items and reasons for revision

**First Version**	**Revised Version**	**Reason(s) for the Revision**
My parents had enough time for me.	My parents spent enough time with me.	Many of the children interpreted the question as the actual amount of time their parents spent with them – half of them revised the questions to "spend time" rather than "had enough time."

I was able to rely on my friends.	I was able to count on my friends.	Some of the children used words like "trust" or "count on" to interpret the question. Two out of six of the children said they weren't sure of the meaning of "rely."

I felt socially accepted by other kids.	I felt accepted by other kids my age.	One of the children didn't know what "socially" meant, but understood the question with it left out.

I did things with other boys and girls.	I did things with kids my age.	All children found the question to be clear and considered both sexes when answering it. However, some defined their interactions with the opposite sex differently than that of their own – it seemed like since the question mentioned the sexes independently it divides the incidence of "doing things" with other children. (I play sports with boys every afternoon. I sometimes play with the girls in gym).

I had enough time to meet friends.	I had enough time to be with my friends.	Three out of six of the children interpreted this question as having time to spend with current friends, two interpreted this as having the time and opportunity to meet new friends, and one child didn't know what this meant. There was an obvious difference in interpretation because of the word "meet."

I felt like I did everything badly.	I felt like I couldn't do anything right	Two of the children interpreted this as meaning doing something that wasn't good enough, while two others interpreted it as doing something "bad" that was worthy of punishment., and the remaining children defined it as "feeling bad" and "my life has been bad." There was a significant degree of difference in interpretation because of the word "badly."

How severe was your asthma?	My asthma was really bad.	Four out of six of the children had a difficultly defining "severe" and three out of six suggested rewording it to "How bad is your asthma."

Did you feel that you got easily exhausted?	I tired easily because of my asthma.	Three out of six of the children had trouble defining or understanding the word "exhausted" and used tired as a synonym to interpret the question.

Did asthma bother you if you wanted to go out?	My asthma bothered me when I was with my friends.	Four out of six of the children defined "go out" as going outside to do something or to play outside. This resulted in some of the children factoring the weather into the state of their asthma. Another kid interpreted "go out" as going to dinner or doing anything else outside of the house. The interpretation was not consistent and if factoring in weather, the degree of variability is even higher.

Did you feel terrible when you were out of breath?	My body felt bad when I was out of breath.	Some children thought that "feeling terrible" was equivalent to feeling guilty after doing something wrong.

Were you scared that you might have to go to the ER?	I was scared that I might have to go to the emergency room or hospital because of my asthma.	One young child didn't know what ER meant.

I could use a mouse for the computer.	I could use a mouse or touch pad for the computer.	One child mentioned that he never used a mouse, but did use a touch pad. Both should be referred to since many laptop users may not use a mouse.

I could drink without help.	I could lift a cup to drink.	* Item revised by project personnel for consistency with other similar items

I could undo snaps.	I could zip up my clothes.	Three out of five of the children weren't sure what the "snaps" were or what the question was referring to. Some thought it was referring to snaps on clothes, while others weren't sure (example – snapping fingers.)

I could turn pages.	I could turn pages in a book.	All of the children mentioned books or magazines when describing the meaning of the question. Two out of five of the children recommended rewording the question to include "turn pages in a book."

I used a special built-up pencil to write.	I used a pencil with a special grip to write.	Many were confused about what a "built-up pencil" is. One defined it as a thick pencil, another thought it was a bendable pencil. However, three out of the five mentioned that they thought it maybe referring to a pencil grip – indicating that it is likely a better descriptor.

I could walk to the bathroom.	I could walk across the room.	Two out of five of the children interpreted the question as being able to find the bathroom and another child referenced going to a bathroom while attending an athletic event in a stadium.

I felt good about my relationship with classmates.	I felt good about how I got along with classmates.	Two out of five of the children said that "relationship" is too hard to understand. A few of the children re-worded it as meaning "to get along" with others.

I worried about my relationships with friends.	I worried about losing a friendship.	Some of the children thought the word "relationship" was too difficult. Also, they interpreted the statement differently. One child thought it meant to be concerned about someone (for their safety or wellbeing), and another thought it meant feeling the need to impress them.

I argued with other kids.	I got into a yelling fight with other kids.	Two out of five of the children recommended not using the word "argue." Three of the five children re-worded the question using the words "yelling" or "fighting."

I felt bad about my relationships with classmates.	I felt bad about how I got along with classmates.	The word "relationship" was dropped because some thought it was too difficult to understand. They also interpreted it differently; one thought it meant to feel bad after arguing, another thought it meant not liking or "feeling good" about classmates.

I got anxious when I went to bed at night.	I worried when I went to bed at night.	Two out of five of the children weren't sure what anxious meant and recommended using a different word.

## Results

Children who participated in the cognitive interviews spent approximately 1 hour with each interviewer, with some children (for example, younger children who took breaks) requiring additional time. In general, even children as young as 8 could understand the majority of the items (293/318 = 92%) and response options, indicating that they could think about and discuss their own health. Although younger children had a more difficult time with specific words, they understood the purpose of the items and response options and were able to provide alternatives using their own vocabulary. They also had no difficulty understanding that they needed to answer questions while thinking about specific recall periods. Older children seemed to clearly understand the majority of items and response options, and had fewer comprehension difficulties than younger children.

Tables 4 and 5 outline common issues identified by participants. Specific words (i.e., irritable, stressed) were difficult to comprehend for some children and items were sometimes too vague or ambiguous to be clearly understood. The majority of items (92%) were retained in the item banks for further large scale testing.

There was no indication that children had difficulty with the response options, except that younger children seemed to misunderstand the word "difficulty". When questioned, children were able to distinguish between the different response options, indicating that they could clearly identify variable levels of functioning, so the word "difficulty" was changed to "trouble" in subsequent cognitive interviews. Additionally, 48/53 (91%) of the children reported that the 7 day recall period meant the previous 7 days, and they responded to items accordingly. A subset of children were probed on present and past tense preferences for the item stems; 8 preferred the present tense, 8 preferred the past tense, and 9 had no stated preference when referring to the past 7 days. Participants had an overall positive opinion of the items and did not provide any suggestions for additional content that was not included in the current item banks.

## Discussion

These results confirm that children ages 8–17 can talk about and respond to items asking them about their health and well-being. They can also offer unique insight into the understandability of the items. These findings are consistent with other studies [5,6]. The majority of the items were well comprehended by all age groups, but we also identified several terms that were not well understood by younger children. Items containing difficult words or vague concepts were readily identified by the children and led to important questionnaire changes.

We also received valuable feedback on the format of the questionnaire, including increasing the font size for ease of readability, shortening the instructions, and putting the recall period in bold type. For some children, certain items were not applicable to them; for example, one child didn't have a computer at home, so he could not answer items related to computer use. Similarly, items that asked about walker or wheelchair use were not applicable to the majority of children interviewed, so feedback was limited for these items.

The sample included an almost equal distribution of children in different age groups, and represented a diverse population. One benefit of the sample is that it included a number of children with asthma, ensuring that comments from children with the most common chronic disease in the United States were included. The sample was well balanced for socioeconomic status and race/ethnicity, which is a strength of this study.

Our study is similar to other cognitive interview studies for children's PRO instrument development. For example, we found that younger children had more difficulty understanding specific item words than older children, particularly for words such as "irritable", "nervous" and "worried". Children in our study also had difficulty understanding ambiguous terms or phrases such as "did things" and "activities". These findings are consistent with other studies of child-reported health outcomes [5,18,19]. Additionally, like other studies, the children in our study reported few issues with the response formats using up to 5 response options, and were able to respond to items within the recall period [5]. On occasion, the PROMIS pediatrics item development team had to decide what to do if a suitable synonym or content description was not available for substitution when a word was not well understood by some children. For example, the idea of "worry" is important content for the anxiety domain and it was kept in the item bank even though some children noted problems. These items will be reviewed again after large scale testing is completed and final decisions for these items will be made at that time.

Our study has several limitations. First, each item received a minimum of 5 cognitive interviews. Although we felt this was sufficient, some authors suggest that 10 – 15 interviews are better [9]. Because of experience on previous scale development projects [5,18,19] with very similar items we felt comfortable performing fewer overall interviews on these items. Since a minimal number of children ages 8 or 9 were required to review the items, some important findings for this age group could be missed. Secondly, as with any qualitative study, the item development team had to make judgments as to the importance of an item problem and whether revisions were necessary. We tried to adhere to the operationalization of two negative comments leading to revision, but all such judgments are inherently qualitative. Our team, however, was interested in identifying the most clear and important items for inclusion and carefully responded to all of the feedback from the children. Lastly, the interview questions about content validity were phrased very broadly and did not add additional information to our previous studies utilizing focus groups [4].

## Conclusion

Overall, the findings of the cognitive interviews suggest that children as young as 8 years could respond to items and talk about all aspects of their health and well-being in meaningful ways. They are able to comprehend varying response options on a categorical scale, and can accurately respond to items using a 7-day recall period. Feedback from the children who participated was valuable in creating a set of items to be administered to a wide age range of children. The final item set generated as a result of the cognitive interview process is currently undergoing large scale testing as part of the PROMIS Pediatric Item Bank development process.

## Abbreviations

(PROMIS): Patient Reported Outcomes Measurement Information System; (PROs): Patient-reported outcomes; (S&W): Scott and White; (UNC): University of North Carolina; (WRAT): Wide Range Achievement Test-3 ReadingSubtest; (PedsQL™): Pediatric Quality of Life Inventory™.

## Competing interests

The authors declare that they have no competing interests.

## Authors' contributions

All authors have made substantial contributions to conception and design, or acquisition of data, or analysis and interpretation of data, been involved in drafting the manuscript or revising it critically for important intellectual content; and have given final approval of the version to be published.
